# Survival after curative hepatectomy for hepatocellular carcinoma in patients with and without MAFLD: a western cohort study

**DOI:** 10.3389/fonc.2026.1793271

**Published:** 2026-07-16

**Authors:** Michele Molinari, Vrishketan Sethi, Christof Kaltenmeier, Abiha Abdullah, Berkay Demirors, Matthew Yu-Sheng Lin, Jason Mial-Anthony, Dooman Arefan, Shandong Wu, Christopher Buros, Hao Liu, Charbel Elias, Marta Minervini, Samer Tohme, Xingyu Zhang, David Geller, Alessandro Furlan

**Affiliations:** 1 Department of Surgery, University of Pittsburgh Medical Center, Pittsburgh, PA, United States; 2 Department of Surgery, Kaohsiung Medical University, Kaohsiung, Taiwan; 3 University of Pittsburgh, Pittsburgh, PA, United States; 4 Department of Surgery, Houston Methodist Hospital, Houston, TX, United States

**Keywords:** adjusted hazard ratio, Cox regression analyses, hepatic resection, hepatocellular carcinoma, metabolic associated fatty liver disease, survival

## Abstract

**Background:**

Evidence suggesting that hepatocellular carcinoma (HCC) arising in patients with metabolic-associated fatty liver disease (MAFLD) is associated with better outcomes than HCC related to other liver diseases has been derived largely from non-Western cohorts and has incompletely accounted for the severity of underlying liver disease. Whether MAFLD independently influences survival after hepatic resection, particularly in Western populations, remains uncertain.

**Methods:**

In this retrospective single-center cohort study, adults who underwent curative-intent hepatic resection for HCC at a Western academic center were evaluated. Patients managed without surgery were excluded. MAFLD was defined according to international consensus criteria. In the primary analysis, patients were classified as MAFLD-positive if they fulfilled MAFLD criteria and had no competing chronic liver disease etiology. Overall survival (OS) and disease-free survival (DFS) were assessed with Cox proportional-hazards models adjusted for cirrhosis, albumin-bilirubin grade, tumor burden, performance status, and alpha-fetoprotein level among other confounders.

**Results:**

Among 156 patients, 89 (57.1%) were classified as MAFLD-positive. As compared with MAFLD-negative patients, MAFLD-positive patients were older, more frequently obese and diabetic, and less likely to have cirrhosis (all P ≤ 0.043). Open or hybrid resection was more common in the MAFLD-positive group, whereas minimally invasive resection was more common in the MAFLD-negative group (67.4% vs. 44.8% and 32.6% vs. 55.2%, respectively; P = 0.006). In unadjusted analyses, MAFLD-positive status was associated with longer DFS and a trend towards longer OS; however, these associations were attenuated after multivariable adjustment. MAFLD was not independently associated with OS (adjusted hazard ratio, 0.91; 95% confidence interval [CI], 0.45 to 1.84; P = 0.785) or DFS (adjusted hazard ratio, 0.58; 95% CI, 0.28 to 1.19; P = 0.139). Cirrhosis, impaired performance status, elevated alpha-fetoprotein, and greater tumor burden were the principal predictors of postoperative outcomes.

**Conclusions:**

In this Western resection cohort, MAFLD was not independently associated with OS or DFS after curative hepatectomy once liver disease severity and tumor burden were taken into account. Prognosis was driven primarily by established hepatic and oncologic factors rather than metabolic etiology alone.

## Introduction

Hepatocellular carcinoma (HCC) is the most common primary liver malignancy and a leading cause of cancer-related death worldwide (1). Although its highest incidence remains in Asia and sub-Saharan Africa, the epidemiology of HCC in Europe and North America has changed substantially over the past two decades (1–4). Declining rates of viral hepatitis–related HCC, driven by widespread hepatitis B vaccination and effective antiviral therapy for hepatitis C, have coincided with a marked rise in metabolic-associated fatty liver disease (MAFLD) as a major etiologic contributor to HCC in Western populations (3, 5–8).

In contrast to viral- and alcohol-related liver disease, MAFLD-associated HCC often arises in the absence of cirrhosis and in patients with relatively preserved hepatic function (8–10). Emerging evidence further suggests that MAFLD-associated HCC may be characterized by distinct oncogenic and inflammatory signaling pathways, raising the possibility that its biological behavior may differ, at least in part, from that of HCC arising from other liver diseases. Several observational studies have compared outcomes after curative-intent hepatic resection in patients with MAFLD-associated HCC and those with HCC related to other etiologies, but the results have been inconsistent (11–21). Two recent systematic reviews and meta-analyses, including 15 and 17 observational studies, respectively, reported improved overall survival (OS) and disease-free survival (DFS) among patients with MAFLD-associated HCC undergoing hepatic resection (22, 23). These findings, however, warrant cautious interpretation. Only four of the included studies were conducted in Europe (17, 24–26), and only two originated from North America (27, 28). Moreover, most studies did not adequately adjust for key determinants of postoperative outcome, including cirrhosis severity, liver functional reserve, and tumor burden (22, 23).

Subgroup analyses from both meta-analyses further suggested that the apparent survival advantage associated with MAFLD was largely confined to Asian cohorts, whereas no significant differences were observed in studies from Western centers (22, 23). Differences in patient selection, background liver disease, tumor presentation, and regional practice patterns may have contributed to these geographic discrepancies. Whether MAFLD independently influences survival after hepatic resection in Western populations therefore remains unresolved.

Given the growing burden of MAFLD-associated HCC in Europe and North America, resolving this question is clinically important. The present study examined a retrospective cohort of consecutive patients with resectable HCC who underwent curative-intent hepatic resection at a high-volume academic center in the United States. The objective was to determine whether the survival differences previously attributed to MAFLD remained after rigorous adjustment for cirrhosis, liver functional reserve, and tumor burden (22, 29).

## Methods

### Study design and population

This retrospective cohort study included consecutive adult patients who underwent curative-intent hepatic resection for hepatocellular carcinoma (HCC) at a high-volume academic center in the United States between January 2011 and December 2020. Follow-up was completed through December 31, 2023. The study was approved by the Institutional Review Board (PRO 13060220), with a waiver of informed consent because of its retrospective design and use of de-identified data. All procedures were conducted in accordance with the Declaration of Helsinki (30) and are reported in accordance with the STROBE statement (31).

### Inclusion and exclusion criteria

Eligible patients were 18 years of age or older and had histologically confirmed HCC treated with hepatic resection. Only patients who underwent hepatic resection for HCC alone were included. Patients with mixed hepatocellular-cholangiocarcinoma, synchronous non-HCC malignancies, prior hepatic resection, prior liver transplantation, or non-curative surgery (R2 resection) were excluded. Patients who received preoperative chemotherapy, locoregional therapy, or immunotherapy remained eligible provided that they ultimately underwent curative-intent hepatic resection.

### Definition of MAFLD

MAFLD was defined according to international consensus criteria, requiring evidence of hepatic steatosis on imaging or histology in the presence of metabolic dysfunction, including overweight or obesity, type 2 diabetes mellitus, or metabolic dysregulation (32, 33). HCC arising in patients with cryptogenic cirrhosis and a history of metabolic dysfunction was also classified as MAFLD-associated (32, 33). To reduce etiologic misclassification in the primary analysis, patients classified as MAFLD-positive did not have other identifiable chronic liver disease etiologies. This approach was selected to improve internal consistency within a limited surgical cohort, while acknowledging that etiologic overlap is common in clinical practice. Patients with HCC arising in the setting of viral hepatitis, alcohol-associated liver disease, autoimmune liver disease, hemochromatosis, or other non-metabolic etiologies were classified as MAFLD-negative.

### Data collection

Demographic, clinical, laboratory, and operative data were extracted from a prospectively maintained institutional registry. Variables included age, sex, body mass index, diabetes, American Society of Anesthesiologists classification (34), Eastern Cooperative Oncology Group performance status (35), and the presence of cirrhosis. Tumor-related variables included tumor size, number, differentiation, angiolymphatic invasion, T stage, and serum alpha-fetoprotein level. Liver functional reserve was assessed using serum albumin, total bilirubin, and the Albumin-Bilirubin (ALBI) score (36).

### Histopathology

All available non-tumoral liver parenchyma was re-reviewed by a single expert liver pathologist (M. Minervini), who was blinded to clinical outcomes. Fibrosis and cirrhosis were assessed using standard histologic criteria based on the Ishak and METAVIR scoring systems (37–40). Cirrhosis was defined as Ishak stage 6 or METAVIR F4.

### Outcomes and follow-up

The primary outcomes were overall survival (OS), defined as the interval from hepatic resection to death from any cause, and disease-free survival (DFS), defined as the interval from resection to radiographic recurrence or death. Secondary outcomes included 90-day mortality, 1-year mortality, intensive care unit admission, length of hospital stay, and 90-day readmission. Postoperative surveillance followed institutional practice aligned with international guidelines (41), including cross-sectional imaging and alpha-fetoprotein measurement every 3 to 4 months for the first 3 years and every 6 months thereafter.

### Statistical analysis

Continuous variables were assessed for normality using the Shapiro-Wilk test and visual inspection of their distributions. Parametric data are presented as means with standard deviations and were compared using Student’s *t*-test or analysis of variance, as appropriate. Nonparametric data are presented as medians with interquartile ranges and were compared using the Mann-Whitney *U* test or Kruskal-Wallis test. Categorical variables were compared using the chi-square test or Fisher’s exact test.

OS and DFS were estimated using the Kaplan-Meier method and compared with the log-rank test. Cox proportional-hazards models were used to examine the independent association between MAFLD status and postoperative outcomes after adjustment for clinically relevant hepatic and oncologic covariates, including cirrhosis, ALBI grade, performance status, alpha-fetoprotein level, and tumor burden. The proportional-hazards assumption was evaluated using Schoenfeld residuals and log-minus-log plots.

To further examine whether the apparent association between MAFLD and survival was influenced by differences in background liver disease, an exploratory sequential modeling strategy was performed. First, the association between MAFLD status and cirrhosis was assessed using multivariable logistic regression adjusted for age and sex. This step was undertaken to determine whether MAFLD status was independently associated with cirrhosis, the background liver characteristic considered most likely to contribute to the crude survival differences observed between groups. Sequential Cox models were then constructed for OS and DFS to evaluate the extent to which the association between MAFLD and postoperative outcomes was attenuated after progressive adjustment for cirrhosis, ALBI grade, and tumor burden. Model 1 included MAFLD status alone; Model 2 included MAFLD status and cirrhosis; Model 3 included MAFLD status and ALBI grade; Model 4 included MAFLD status, cirrhosis, and ALBI grade; and Model 5 additionally included tumor burden variables.

Formal mediation analysis was not performed because this retrospective surgical cohort was not optimally suited for causal mediation modeling. Specifically, the modest sample size, the selected nature of the study population, and the time-to-event structure of the endpoints would have required assumptions that could not be adequately verified, including the absence of unmeasured exposure-mediator and mediator-outcome confounding. Accordingly, the sequential models were interpreted as exploratory attenuation analyses rather than causal mediation analyses.

All tests were two-sided, and a *P* value of less than 0.05 was considered to indicate statistical significance. Analyses were performed using IBM SPSS Statistics, version 31.0.2.0 (IBM Corp., Armonk, NY, USA).

### Power and sample size considerations

Power calculations for Cox regression analyses were based on a two-sided alpha of 0.05 and 80% power, using effect sizes derived from prior meta-analyses (pooled OS hazard ratio, 0.78; pooled DFS hazard ratio, 0.81) and an anticipated MAFLD prevalence of 57% (22). Detecting effects of this magnitude would require approximately 800 to 1,000 patients for OS and 900 to 1,100 patients for DFS, whereas detection of the smaller Western OS effect size (hazard ratio, 0.85) would require more than 1,700 patients. The present study was therefore not powered to detect small MAFLD-specific effects and should be interpreted as an exploratory single-center surgical cohort analysis. The principal analytic objective was to determine whether survival differences observed in unadjusted analyses persisted after adjustment for established hepatic and oncologic prognostic factors rather than to infer the absence of small independent effects.

## Results

### Baseline characteristics

A total of 156 adult patients met the inclusion criteria, including 89 (57.1%) classified as MAFLD-positive and 67 (42.9%) classified as MAFLD-negative. Baseline demographic and clinical characteristics are summarized in **Table 1**. Compared with MAFLD-negative patients, MAFLD-positive patients were older (70.1 ± 9.0 vs. 67.4 ± 9.5 years; *P* = 0.043), had a higher body mass index (31.4 ± 5.6 vs. 26.6 ± 5.5 kg/m²; *P* < 0.001), and had a higher prevalence of diabetes (65.2% vs. 32.8%; *P* < 0.001). Cirrhosis was substantially less common in the MAFLD-positive group than in the MAFLD-negative group (27.0% vs. 64.2%; *P* < 0.001).

**Table 1:** Characteristics of the cohort of patients with MAFLD-positive versus MALFD-negative HCC.

Characteristics, Total N. 156	MAFLD-positive	MAFLD-negative	*P Value*
	n. 89	n. 67	
Age, years, mean, (SD)	70.1 (9.0)	67.4 (9.5)	**0.043**
Sex, male/female, n.	64/25	50/17	0.720
Body mass index, mean, (SD)	31.4 (5.6)	26.6 (5.5)	**<0.001**
Body mass index category, n. (%)			
Non-obese	40 (44.9)	55 (82.1)	**<0.001**
Obese class I	26 (29.2)	7 (10.4)	
Obese class II	17 (19.1)	3 (4.5)	
Obese class III	6 (6.7)	2 (3.0)	
History of diabetes, n. (%)	58 (65.2)	22 (32.8)	**<0.001**
History of cigarette smoking, n. (%)	57 (64.8)	44 (65.7)	0.908
American Society of Anesthesia (ASA) classification, n. (%)			
ASA I	0	0	0.640
ASA II	7 (7.9)	4 (6.0)	
ASA III	66 (74.2)	54 (80.6)	
ASA IV	16 (18.0)	9 (13.4)	
ASA V	0	0	
Liver Parenchyma			
Normal	15 (16.9)	7 (10.4)	**<0.001**
Fibrosis	50 (56.1)	17 (19.5)	
Cirrhosis	24 (27.0)	43 (64.2)	
Model for end-stage liver disease (MELD), mean (SD)	5.7 (3.6)	6.9 (4.7)	0.077
Surgical approach, n. (%)			
Open or hybrid hepatic resection	60 (67.4)	30 (44.8)	**0.006**
Minimally invasive hepatic resection	29 (32.6)	37 (55.2)	
Operative time, minutes, mean (SD)	200.9 (156.3)	152.9 (100.8)	**0.030**
Type of resection, n. (%)			
Left lateral resection (segments II and III)	10 (11.2)	2 (3.0)	**0.003**
Left lobectomy (segments II, III, IV)	4 (4.5)	3 (4.5)	
Extended left hepatectomy (segments II, III, IV, V, VIII)	4 (4.5)	3 (4.5)	
Right lobectomy (segments V, VI, VII, VIII)	4 (4.5)	1 (1.5)	
Extended right hepatectomy (segments V, VI, VII, VIII, IV)	8 (9.0)	0 (0)	
Resection of I segment	18 (20.2)	31 (46.3)	
Resection of II segments	27 (30.3)	24 (35.8)	
Resection of III or more non-adjacent segments)	14	3 (4.5)	
Preoperative platelet count, mean, (SD)	200,494 (104.455)	168,089 (98,961)	0.051
Preoperative serum albumin, g/dL, mean (SD)	3.61 (0.5)	3.6 (0.5)	0.671
Preoperative serum total bilirubin, mg/dL, mean (SD)	0.85 (0.7)	0.91 (0.6)	0.555
Preoperative serum creatinine, mean (SD)	0.97 (0.3)	1.04 (0.7)	0.410
Preoperative serum creatinine above 1.5 mg/dL, n. (%)	9 (10.1)	5 (7.6)	0.778
Preoperative International Normalized Ratio (INR), mean (SD)	1.1 (0.2)	1.2 (0.4)	0.052
Intensive Care Unit (ICU) admission, n. (%)	17 (19.1)	6 (9.0)	0.060
Length of hospital stay, days, mean, (SD)	5.9 (6.1)	4.4 (4.6)	0.093
Hospital readmission within 90-days	16 (18.0)	21 (31.3)	0.233

Bold means statistically significant differences between the two groups.

The operative approach also differed between groups. Open or hybrid hepatic resection was more common among MAFLD-positive patients, whereas minimally invasive hepatic resection was more common among MAFLD-negative patients (67.4% vs. 44.8% and 32.6% vs. 55.2%, respectively; *P* = 0.006). Mean operative time was longer in MAFLD-positive patients (200.9 ± 156.3 vs. 152.9 ± 100.8 minutes; *P* = 0.030). The proportions of missing baseline data are reported in **Supplementary Table 1**.

### Oncologic characteristics

Oncologic characteristics are summarized in **Table 2**. MAFLD-positive patients had larger tumors than MAFLD-negative patients (mean diameter, 5.6 cm vs. 3.8 cm; *P* = 0.004). Bilobar disease was more frequent in the MAFLD-positive group, although this difference did not reach statistical significance (29.2% vs. 20.9%; *P* = 0.417). Tumor differentiation, angiolymphatic invasion, Eastern Cooperative Oncology Group performance status, and alpha-fetoprotein distribution were otherwise similar between groups. The extent of missing oncologic data is shown in **Supplementary Table 2**.

**Table 2:** Oncological characteristics: MAFLD-positive versus MAFLD-negative patients.

Characteristics	MAFLD-positive n.89	MAFLD-negative n.67	*P Value*
Negative resection margins, n. (%)	70 (79.5)	48 (71.6)	0.170
Resection margins < 1 mm from tumor, n. (%)	18 (20.5)	19 (28.4)	
Angiolymphatic invasion, n. (%)	37 (42.0)	28 (41.8)	0.553
Tumor differentiation			
Well differentiated	18 (20.2)	17 (25.4)	0.834
Moderately differentiated	57 (64.0)	42 (62.7)	
Poorly differentiated	12 (13.5)	7 (10.4)	
Tumor location			
Bilobar tumors	26 (29.2)	14 (20.9)	0.417
Right lobe	33 (37.1)	31 (46.3)	
Left lobe	29 (32.6)	21 (31.3)	
Caudate lobe	1 (1.1)	1 (1.5)	
T stage			
1a	31 (34.8)	28 (41.7)	0.193
1b	6 (6.7)	2 (2.9)	
2	36 (40.4)	25 (37.3)	
3a	8 (8.9)	2 (2.9)	
3b	3 (3.3)	1	
4	3 (3.3)	5	
Diameter of largest tumor, cm, mean, (SD)	5.6 (3.9)	3.8 (3.3)	**0.004**
Number of tumors, mean (SD)	1.7 (1.2)	1.6 (1.1)	0.334
1	54 (60.7)	42 (62.7)	0.574
2	20 (22.5)	15 (22.4)	
3	8 (9.0)	6 (9.0)	
4	3 (3.4)	0 (0)	
5	2 (2.2)	3 (4.5)	
>5	2 (2.2)	1 (0.6)	
ECOG, n. (%)			
ECOG 0	46 (61.3)	33 (61.1)	0.572
ECOG 1	15 (20.0)	14 (25.9)	
ECOG ≥2	14 (18.7)	7 (13.0)	
Unknown	14	13	
Alpha feto-protein (AFP), median, IQR	876.9 (5,704)	3,360 (15,954)	0.183
AFP category			
AFP 0-10	56 (65.1)	38 (56.7)	0.674
10.1-100	18 (20.9)	18 (26.9)	
>100	12 (13.9)	11 (16.5)	
Neutrophil-to-Lymphocyte Ratio (NLR), mean (SD)	4.1 (5.0)	3.1 (1.9)	0.102
Neutrophil-to-Lymphocyte Ratio (NLR) >3, n. (%)	42 (47.2)	25 (37.3)	0.254
Platelet-to-Lymphocyte Ratio (PLR), mean (SD)	156.1 (119.2)	132.1 (102.1)	0.186
Platelet-to-Lymphocyte Ratio (PLR) > 150, n. (%)	32 (36.0)	16 (23.9)	0.118
ALBI score, n. (%)			
Grade 1	32 (37.2)	21 (32.3)	0.811
Grade 2	46 (53.5)	38 (58.5)	
Grade 3	8 (9.3)	6 (9.2)	

Bold means statistically significant differences between the two groups.

### Unadjusted survival analysis

**Figure 1:**
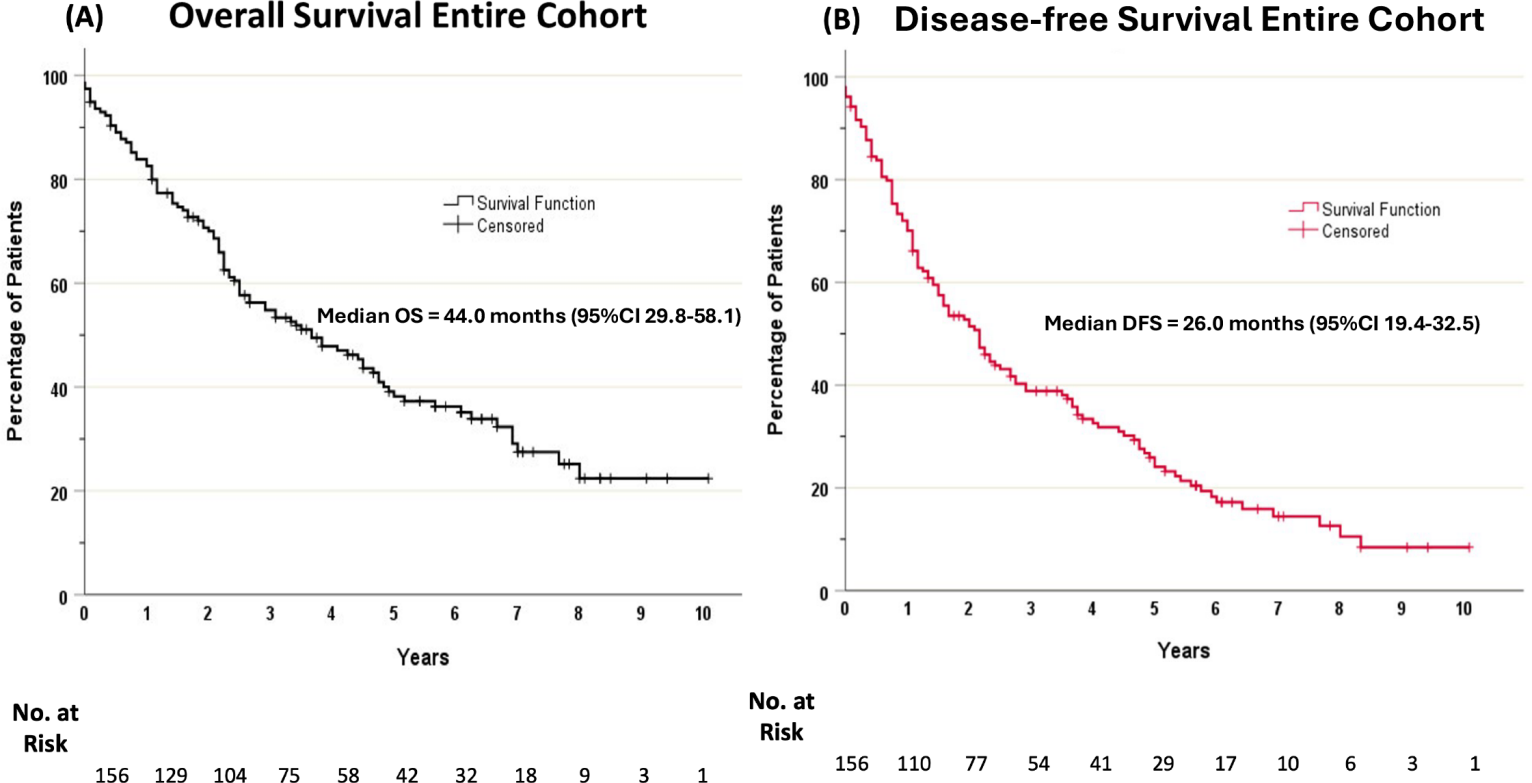
Kaplan-Meier survival curves depicting overall survival (OS) **(A)** and disease-free survival (DFS) **(B)** in a consecutive cohort of patients undergoing curative-intent hepatic resection for hepatocellular carcinoma.

**Figure 2:**
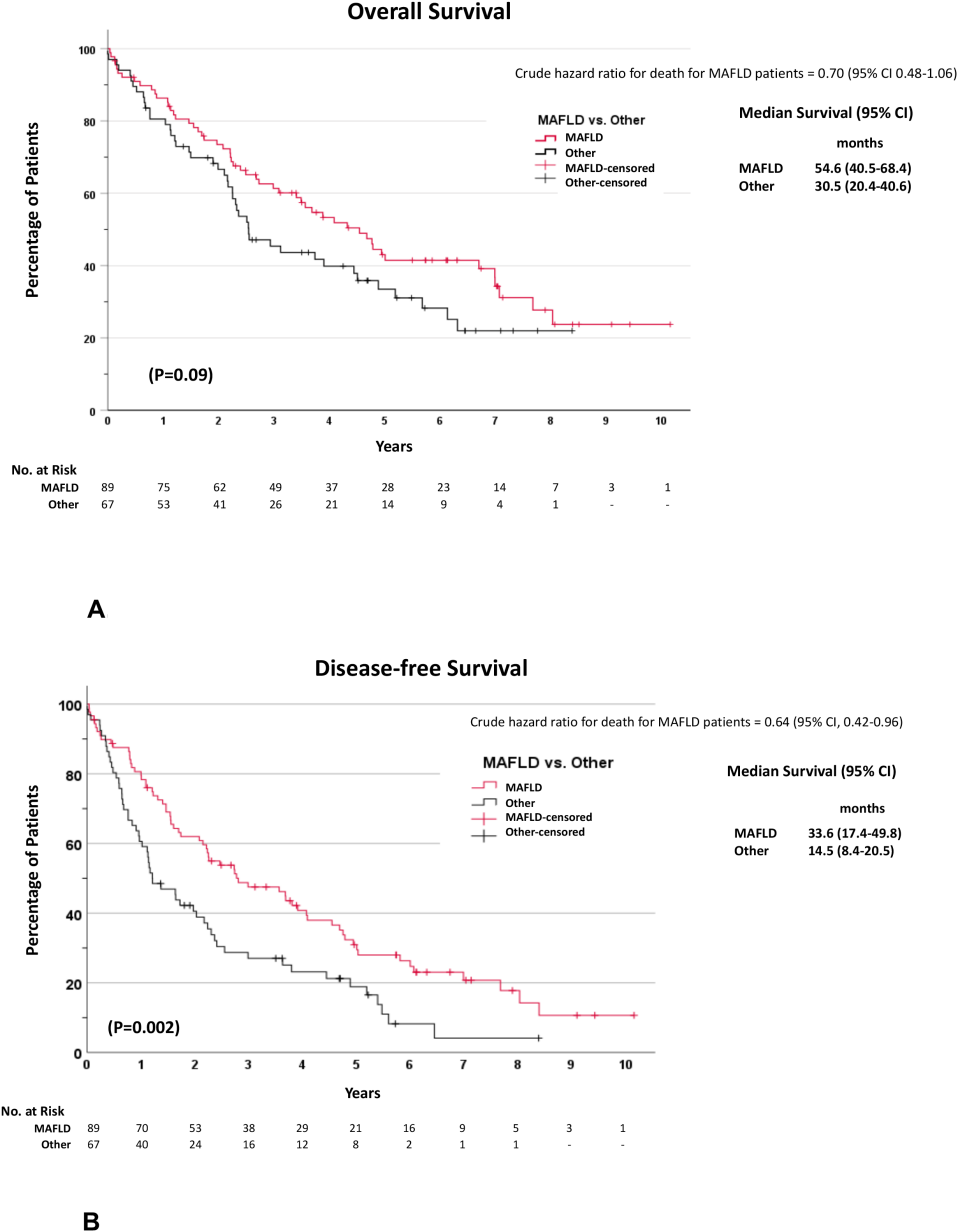
**(A)** Kaplan-Meier curves illustrating overall survival (OS) following hepatic resection for hepatocellular carcinoma in patients with metabolic-associated fatty liver disease (MAFLD-positive) compared to those with HCC related to non-MAFLD etiologies (MAFLD-negative). **(B)** Kaplan-Meier curves illustrating disease-free survival (DFS) following hepatic resection for hepatocellular carcinoma in patients with metabolic-associated fatty liver disease (MAFLD-positive) compared to those with HCC related to non-MAFLD etiologies (MAFLD-negative).

Median follow-up was 44.0 months (95% confidence interval [CI], 29.8 to 58.1) (**Figure 1**). Ninety-day mortality occurred in 11 patients (7.1%), and 1-year mortality in 25 (16.0%), with no significant between-group differences (90-day mortality, 7.9% vs. 6.0%, *P* = 0.759; 1-year mortality, 13.5% vs. 19.4%, *P* = 0.380). Kaplan-Meier curves for overall survival (OS) and disease-free survival (DFS) are shown in **Figures 2A, B**, respectively. Five-year OS showed a nonsignificant trend toward improvement in the MAFLD-positive group (*P* = 0.09), whereas DFS was significantly longer among MAFLD-positive patients (*P* = 0.002).

### Multivariable survival analysis

After adjustment for clinically relevant covariates, MAFLD status was not independently associated with OS (adjusted hazard ratio [HR], 0.91; 95% CI, 0.45 to 1.84; *P* = 0.785) or DFS (adjusted HR, 0.58; 95% CI, 0.28 to 1.19; *P* = 0.139). Independent predictors of worse OS included cirrhosis (HR, 2.32; 95% CI, 1.16 to 4.64; *P* = 0.018), Eastern Cooperative Oncology Group performance status ≥2 (HR, 2.67; 95% CI, 1.29 to 5.50; *P* = 0.008), and serum alpha-fetoprotein >100 ng/mL (HR, 2.89; 95% CI, 1.45 to 5.76; *P* = 0.003) (**Table 3**). For DFS, independent predictors of adverse outcome included multiple tumors and serum alpha-fetoprotein >100 ng/mL (**Table 4**).

**Table 3:** Univariate and multivariate analysis of risk factors for overall survival (OS) after radical hepatic resection for hepatocellular carcinoma.

Characteristics	Univariate analysis				Multivariate analysis			
	HR	95% Confidence		P Value	Adjusted HR	95% Confidence		P Value
		LCI	UCI			LCI	UCI	
Diagnosis of liver disease								
MAFLD-negative	1	**Reference**		**-**	1	**Reference**		
MAFLD-positive	0.70	0.48	1.06	0.093	0.91	0.45	1.84	0.785
Presence of cirrhosis	**1.62**	**1.09**	**2.42**	**0.019**	2.32	1.16	4.64	**0.018**
Age	1.03	1.01	1.05	**0.009**	1.02	0.99	1.06	0.223
Sex								
Female	1	**Reference**				**Reference**		
Male	1.22	0.77	1.94	0.400	1.34	0.72	2.48	0.356
Diabetes	0.99	0.67	1.47	0.960	0.98	0.56	1.72	0.938
Cigarette smoking	0.87	0.57	1.33	0.522	0.57	0.32	1.03	0.062
Obesity								
Non-obese	1	**Reference**			1	**Reference**		
Obese	0.663	0.434	1.011	0.056	0.76	0.41	1.40	0.380
ECOG								
ECOG 0	1	**Reference**			1	**Reference**		
ECOG 1	1.35	0.81	2.27	0.253	1.74	0.95	3.18	0.075
ECOG ≥2	1.89	1.11	3.24	**0.019**	2.67	1.29	5.50	**0.008**
Number of tumors								
Single lesion	1	**Reference**			1	**Reference**		
Two lesions	1.22	0.75	1.99	0.418	1.26	0.66	2.40	0.484
Three or more lesions	1.33	0.78	2.26	0.294	1.31	0.61	2.80	0.491
Alpha feto-protein								
0-10	1	**Reference**			1	**Reference**		
10.1-100	1.194	0.729	1.955	0.482	1.39	0.73	2.67	0.319
>100	1.821	1.069	3.100	**0.027**	2.89	1.45	5.76	**0.003**
Cellular differentiation								
Well differentiated	1	**Reference**		**-**	1	**Reference**		–
Moderately differentiated	0.305	0.089	1.042	0.058	0.17	0.01	2.06	0.162
Poorly differentiated	0.469	0.147	1.500	0.202	0.32	0.03	4.13	0.386
Lymphovascular invasion	0.634	0.181	2.232	0.478	1.05	0.52	2.12	0.894
Tumor stage								
T1	1	**Reference**		**-**	1	**Reference**		–
T2	1.086	0.693	1.700	0.719	0.91	0.41	2.00	0.817
T3	1.98	1.055	3.715	**0.033**	2.91	0.92	9.18	0.069
T4	2.464	1.098	5.529	**0.029**	1.91	0.61	6.01	0.269
Neutrophil-to-Lymphocyte Ratio (NLR) >3	1.506	1.012	2.242	**0.043**	1.44	0.83	2.48	0.196
Platelet-to-Lymphocyte Ratio (PLR) > 150	0.861	0.557	1.330	0.499	1.01	0.52	1.96	0.986
ALBI score								
Grade 1	1	**Reference**		**-**	1	**Reference**		–
Grade 2	1.717	1.074	2.746	**0.024**	1.05	0.57	1.92	0.876
Grade 3	2.121	1.017	4.424	**0.045**	0.73	0.27	1.98	0.536

Bold means statistically significant differences between the two groups.

**Table 4:** Univariate and multivariate analysis of risk factors for disease-free survival (DFS) after radical hepatic resection for hepatocellular carcinoma.

Characteristics	Univariate analysis				Multivariate analysis			
	HR	95% Confidence		P Value	Adjusted HR	95% Confidence		P Value
		LCI	UCI			LCI	UCI	
Diagnosis of liver disease				**0.003**				0.845
MAFLD-negative	1	**Reference**			1	**Reference**		
MAFLD-positive	0.57	0.40	0.82	**0.003**	0.58	0.28	1.19	0.139
Presence of cirrhosis	1.69	1.18	2.43	**0.004**	1.82	0.86	3.85	0.117
Age	1.01	0.99	1.03	0.262	0.99	0.95	1.03	0.862
Sex								
Female	1	**Reference**			1	**Reference**		
Male	1.31	0.86	1.99	0.207	1.52	0.70	3.20	0.284
Diabetes	0.97	0.81	1.16	0.753	1.37	0.72	2.61	0.334
Cigarette smoking	1.03	0.70	1.50	0.898	0.59	0.29	1.19	0.144
Obesity	0.641	0.44	0.934	**0.021**	1.10	0.57	2.09	0.773
ECOG				0.655	0.378			
ECOG 0	1	**Reference**			1	**Reference**		
ECOG 1	1.19	0.74	1.92	0.468	1.42	0.69	2.94	0.336
ECOG ≥2	1.21	0.72	2.04	0.462	1.00	0.10	9.52	0.998
Number of tumors				**0.004**	**0.016**			
Single lesion	1	**Reference**			1	**Reference**		
Two lesions	1.86	1.20	2.89	**0.005**	2.34	1.12	4.93	**0.024**
Three or more lesions	1.82	1.14	2.91	**0.012**	3.10	1.27	7.60	**0.013**
Alpha feto-protein				**0.015**				**0.003**
0-10	1	**Reference**			1	**Reference**		
10.1-100	1.44	0.93	2.22	0.100	1.68	0.97	2.93	0.065
>100	2.00	1.21	3.30	**0.007**	3.12	1.59	6.09	**<0.001**
Cellular differentiation				0.857				0.364
Well differentiated	1	**Reference**			1	**Reference**		
Moderately differentiated	0.69	0.21	2.29	0.547	0.72	0.22	1.58	0.898
Poorly differentiated	0.72	0.23	2.30	0.584	0.82	0.24	2.31	0.905
Lymphovascular invasion	1.07	0.75	1.54	0.697	0.58	0.26	1.29	0.184
Tumor stage				0.096				0.658
T1	1	**Reference**			1	**Reference**		
T2	1.11	0.74	1.65	0.615	0.74	0.44	1.24	0.249
T3	1.67	0.92	3.02	0.092	1.57	0.71	3.47	0.267
T4	2.23	1.05	4.72	**0.037**	1.80	0.73	4.47	0.204
Neutrophil-to-Lymphocyte Ratio (NLR) >3	1.50	1.04	2.14	**0.028**	1.43	0.94	2.19	0.098
Platelet-to-Lymphocyte Ratio (PLR) > 150	0.77	0.52	1.14	0.189	1.00	0.54	1.85	0.996
ALBI score				**0.012**				0.972
Grade 1	1	**Reference**			1	**Reference**		
Grade 2	1.56	1.04	2.33	**0.032**	1.01	0.59	1.74	0.968
Grade 3	2.55	1.31	4.94	**0.006**	1.11	0.46	2.71	0.817

Bold means statistically significant differences between the two groups.

### Exploratory sequential attenuation analyses

**Figure 3:**
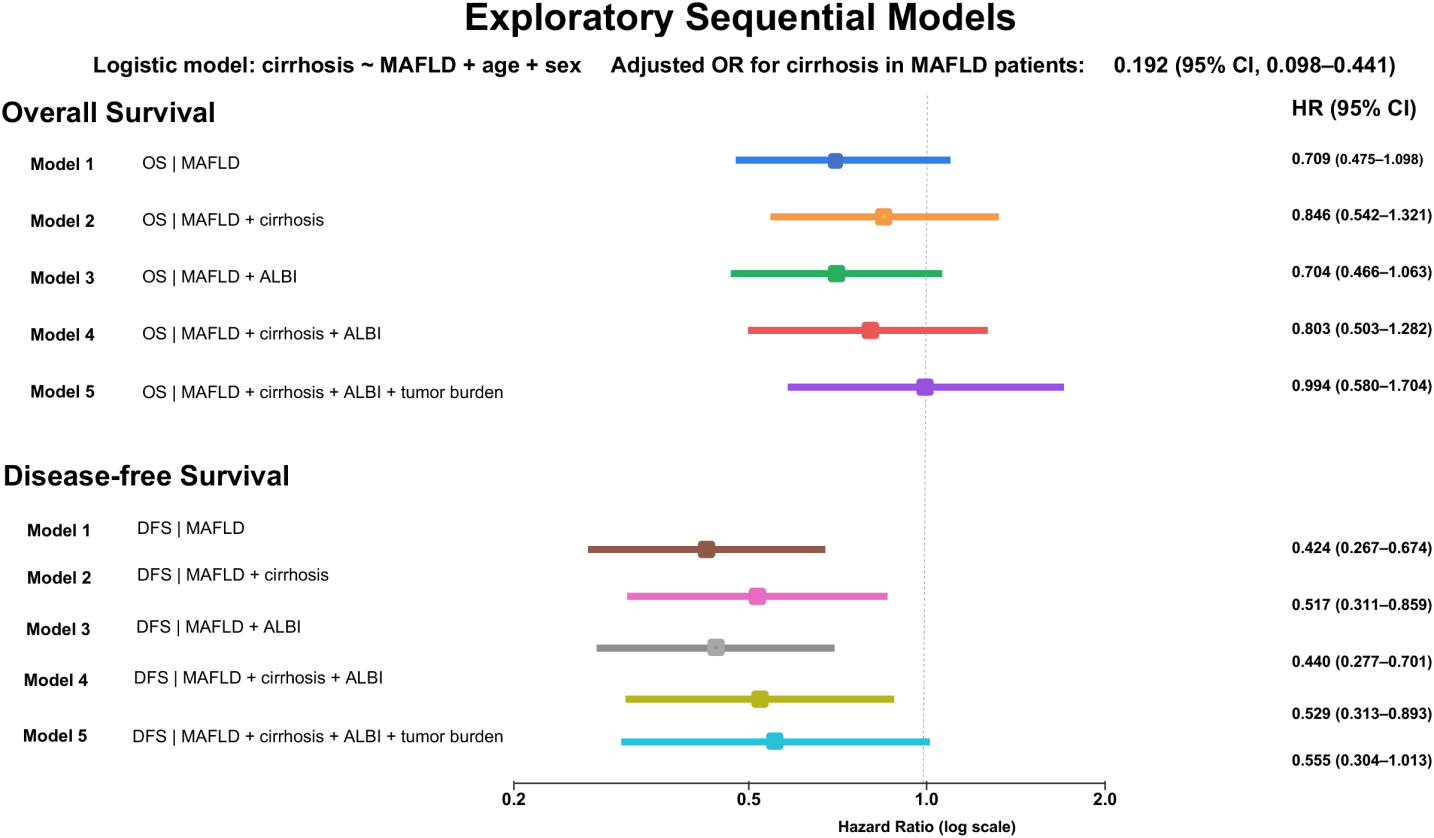
Sequential attenuation of the association between MAFLD status and survival after curative hepatectomy for hepatocellular carcinoma. Sequential Cox models demonstrate that the apparent association between MAFLD status and postoperative overall survival (OS) and disease-free survival (DFS) becomes progressively attenuated after adjustment for cirrhosis, Albumin-Bilirubin (ALBI) grade, and tumor burden, as reflected by hazard ratios moving closer to 1.0 across sequential models. Hazard ratios with 95% confidence intervals are presented for each model.

To further examine whether the crude survival differences associated with MAFLD status were influenced by differences in background liver disease and disease presentation, an exploratory sequential modeling strategy was performed. In multivariable logistic regression adjusted for age and sex, MAFLD-positive patients had significantly lower odds of cirrhosis than MAFLD-negative patients. Sequential Cox models for OS and DFS are shown in **Figure 3**. For OS, the favorable association observed in the unadjusted model was progressively attenuated after sequential adjustment for cirrhosis, ALBI grade, and tumor burden, approaching the null in the fully adjusted model. A similar pattern was observed for DFS, with the initial association between MAFLD and improved outcome becoming progressively weaker after adjustment for background liver parenchymal disease and oncologic burden. Taken together, these exploratory analyses suggest that the crude survival differences associated with MAFLD were more consistent with differences in underlying liver parenchymal disease and oncologic stage at presentation than with an independent prognostic effect of metabolic etiology itself.

## Discussion

The principal finding of this study is that MAFLD status was not independently associated with overall or disease-free survival after curative-intent hepatic resection for HCC once cirrhosis, liver functional reserve, and tumor burden were taken into account. Although MAFLD-positive patients appeared to have more favorable outcomes in unadjusted analyses, those differences diminished after adjustment for established hepatic and oncologic determinants of prognosis. This suggests that the crude survival separation between MAFLD-positive and MAFLD-negative patients was driven primarily by differences in background liver disease and disease presentation rather than by metabolic etiology alone.

This finding helps refine the interpretation of prior studies reporting superior postoperative outcomes among patients with MAFLD-associated HCC (17, 19, 22, 28). Much of that literature has been limited by heterogeneous etiologic definitions, incomplete adjustment for cirrhosis severity and liver reserve, and a relative underrepresentation of Western cohorts (22, 23). In the present study, once cirrhosis, ALBI grade, performance status, AFP, and tumor burden were incorporated into the models, MAFLD no longer contributed independent prognostic information. In this surgical setting, outcome appears to be determined chiefly by hepatic reserve and oncologic burden, which remain the dominant drivers of survival after resection.

The exploratory sequential attenuation analyses add internal support to this interpretation. MAFLD-positive patients had substantially lower adjusted odds of cirrhosis, and the initially favorable association between MAFLD and survival progressively weakened as cirrhosis, ALBI grade, and tumor burden were entered into the models. Although these analyses were not intended to establish formal mediation, they indicate that the apparent prognostic advantage of MAFLD-positive patients is more consistent with less advanced background liver disease and more favorable hepatic reserve than with an intrinsic survival benefit conferred by MAFLD itself. This is an important distinction because it shifts the emphasis from etiology as a prognostic label to the hepatic and oncologic features that more directly determine postoperative outcome.

A second notable finding is the high prevalence of non-cirrhotic disease among MAFLD-positive patients. Fewer than one-third of MAFLD-associated HCC cases occurred in cirrhotic livers, compared with nearly two-thirds of MAFLD-negative cases. This pattern is consistent with the increasingly recognized non-cirrhotic phenotype of MAFLD-associated hepatocarcinogenesis (42–44). In the present cohort, MAFLD-positive patients also presented with significantly larger tumors, yet without more adverse histopathologic features. Taken together, these observations are more compatible with delayed detection than with a more aggressive tumor phenotype.

This finding has direct implications for surveillance. Because current surveillance strategies remain anchored to cirrhosis-based risk stratification, patients with MAFLD and preserved liver function are less likely to be identified early in the course of disease (45–47). The present data reinforce that concern by showing that MAFLD-positive patients were less likely to have cirrhosis but nevertheless came to resection with larger tumors. As the prevalence of MAFLD continues to increase, fibrosis-based surveillance alone is likely to become increasingly insufficient for identifying patients at risk of HCC in Western populations. More effective strategies will likely require integrated models that incorporate metabolic risk factors, liver function, and possibly inflammatory or imaging-derived features to improve early detection and refine postoperative risk stratification (16).

From a surgical standpoint, the results are reassuring. Despite a greater burden of obesity, diabetes, and other metabolic comorbidities, MAFLD-positive patients did not experience worse perioperative outcomes. Perioperative mortality, intensive care utilization, and length of stay were comparable between groups, suggesting that hepatic resection can be performed safely in appropriately selected patients irrespective of underlying liver disease etiology (35, 48). Similarly, the absence of worse pathologic features and the comparable achievement of negative margins indicate that MAFLD status itself should not be viewed as an adverse determinant of resectability or operative intent. Surgical decision-making should therefore remain anchored to hepatic reserve, technical feasibility, and tumor burden rather than to metabolic etiology alone (49–55).

The present study has several important strengths. It evaluated a well-defined Western resection cohort treated at a high-volume academic center, thereby providing data directly relevant to contemporary surgical practice. Centralized histologic reassessment of non-tumoral liver parenchyma by a single expert liver pathologist improved the characterization of fibrosis and cirrhosis, which was central to the study hypothesis. The dataset further included detailed clinical, operative, pathologic, and survival information, allowing adjustment for major hepatic, oncologic, and patient-level covariates. Importantly, the addition of exploratory sequential attenuation analyses strengthened the manuscript by clarifying how much of the crude MAFLD-survival association was explained by differences in liver parenchymal disease and oncologic presentation.

Several limitations should also be acknowledged. First, this was a modest-sized single-center cohort and was not powered to detect small independent survival effects attributable to MAFLD, particularly in Western populations. Accordingly, the present study was well suited to determine whether a large apparent survival advantage persisted after rigorous adjustment, but not to exclude small independent effects with precision.

Second, these limitations are even more relevant for formal mediation analysis. A mediation framework for time-to-event outcomes would have required substantially greater statistical power than was available in the present cohort because it would have depended on stable estimation of the exposure-mediator association, the mediator-outcome association, and the decomposition of total effects into direct and indirect components. In practical terms, such an approach would have required a substantially larger cohort and a higher number of events than were available in this study, particularly given the correlation between cirrhosis and liver reserve measures and the complexity of modeling survival endpoints. Even if technically feasible, any such analysis in this dataset would have been vulnerable to instability and overinterpretation.

Third, the study was intentionally restricted to patients undergoing curative-intent hepatic resection and therefore reflects a selected subgroup with resectable disease rather than the broader HCC population. These findings should not be extrapolated to patients treated with transplantation, locoregional therapies, systemic therapy, or noncurative surgery. Fourth, the primary MAFLD definition excluded patients with competing chronic liver disease etiologies in order to reduce etiologic heterogeneity within a limited cohort. Although this improved internal consistency, it does not fully capture the overlap between metabolic dysfunction and other chronic liver diseases encountered in routine practice. Fifth, portal hypertension was not systematically captured in a standardized manner, limiting more granular assessment of surgical selection. Finally, although formal mediation analysis was not performed primarily for reasons of power and design, its causal interpretation would also have depended on assumptions that could not be adequately verified in this retrospective, surgically selected cohort with time-to-event endpoints, including the absence of unmeasured exposure-mediator and mediator-outcome confounding.

In summary, among patients undergoing curative-intent hepatic resection for HCC, MAFLD status was not independently associated with OS or DFS after adjustment for cirrhosis, liver functional reserve, and tumor burden. The apparent survival advantage observed in unadjusted analyses was explained largely by differences in background liver disease and oncologic presentation. These findings support risk stratification and surgical decision-making based primarily on established hepatic and oncologic factors, while underscoring the need for more effective surveillance strategies for the growing population of patients with non-cirrhotic MAFLD-associated HCC.

## Data Availability

The raw data supporting the conclusions of this article will be made available by the authors, without undue reservation.

## Supplementary material

Supplementary Table 1 Missing variables for the entire cohort of patients

Supplementary Table 2 Missing oncological variables for the entire cohort of patients
