# Supplementary material for: Survival after curative hepatectomy for hepatocellular carcinoma in patients with and without MAFLD: a western cohort study
Source: Front Oncol. 2026 Jul 16;16:1793271. doi: 10.3389/fonc.2026.1793271 (PMC13421399; doi:10.3389/fonc.2026.1793271)
Supplement: Supplementary Table 1 — Missing variables for the entire cohort of patients [file Table1.docx]

| **Supplementary Table 1.** Missing variables for the entire cohort of patients | | |
| --- | --- | --- |
| **Characteristics** | **N. patients with missing data** | **(%)** |
| Age | 0 | 0 |
| Sex | 0 | 0 |
| Primary cause of liver disease | 0 | 0 |
| Body mass index | 0 | 0 |
| History of diabetes | 0 | 0 |
| History of cigarette smoking | 1 | 0.6 |
| American Society of Anesthesia (ASA) classification | 0 | 0 |
| Liver parenchyma quality | 0 | 0 |
| Model for end stage liver disease | 4 | 2.5 |
| Type of surgical procedure | 0 | 0 |
| Operative time | 0 | 0 |
| Hepatic resection | 0 | 0 |
| Albumin | 4 | 2.5 |
| Platelet count before surgery | 0 | 0 |
| Preoperative serum bilirubin | 1 | 0.6 |
| Preoperative serum creatinine | 1 | 0.6 |
| Preoperative INR | 4 | 2.5 |
| Intensive care unit admission | 0 | 0 |
| Perioperative blood transfusions | 0 | 0 |
| Length of hospital stay | 0 | 0 |
| Readmission within 90 days | 1 | 0.6 |
| Alpha-feto-protein (AFP) | 4 | 2.4 |
| Anatomical hepatic resection | 0 | 0 |
| Preoperative platelet count | 2 | 1.2 |
| Serum total bilirubin before surgery | 1 | 0.6 |

| **Supplementary Table 2.** Missing oncological variables for the entire cohort of patients | | |
| --- | --- | --- |
| **Oncological characteristics** | **N. of patients with missing data** | **(%)** |
| Resection margins status | 1 | 0.6 |
| Angiolymphatic invasion | 1 | 0.6 |
| Tumor differentiation | 3 | 1.9 |
| T stage | 2 | 1.2 |
| Number of tumors | 0 | 0 |
| Tumor distribution | 0 | 0 |
| Alpha-feto-protein (AFP) | 4 | 2.4 |
| Diameter of largest tumor | 0 | 0 |
| Neutrophil-to-Lymphocyte Ratio (NLR) | 0 | 0 |
| Platelet-to-Lymphocyte Ratio (PLR) | 0 | 0 |
| ALBI score | 5 | 3.2 |
| ECOG Status | 27 | 17.3 |
